# Supplementary material for: The gut mycobiota of rural and urban individuals is shaped by geography
Source: BMC Microbiol. 2020 Aug 17;20:257. doi: 10.1186/s12866-020-01907-3 (PMC7430031; doi:10.1186/s12866-020-01907-3)
Supplement: Supplementary file 1 — Additional file 1. Questionnaire Details of the questionnaire provided to participants prior to enrolment in the study. The questionnaire details essential required information, clinical information, voluntary dietary information and questions regarding data sharing. [file 12866_2020_1907_MOESM1_ESM.docx]

**Additional File 1 Questionnaire** Details of the questionnaire provided to participants prior to enrolment in the study. The questionnaire details essential required information, clinical information, voluntary dietary information and questions regarding data sharing.

**Questionnaire**

You will have to fill the following questionnaire if you decide to join the study. Please do not complete the section if you are unsure of the required information. The project PI is available to explain all sections.

**Essential information (required)**

*Answering the questions in this section is mandatory and this data will be used anonymously in our network.*

1. What is your birth year? Date: ___/___/19___
2. What is your Weight (in kilograms)? __________
3. What is your gender? ☐Male☐ Female
4. Are you a smoker? If no, are you an ex-smoker? (select one)

☐ Yes ☐No ☐ Ex-smoker

6. What is your usual diet? (select one)

☐ Omnivorous ☐ Vegetarian ☐ Vegan ☐ Other

If you selected “other”please describe here:______________________

7. What is your place of birth? ____________________________________________________________

8. What is your ethnicity? _________________________________________________________________

9. Is your permanent residence rural, urban or mixed? _________________________________________________

10. Do you travel regularly? ☐Yes ☐No

If you selected “Yes” please select the best description for your travel destination:

☐ Urban ☐ Rural ☐ Mixed

11. In case we identify microbiota subsets that have been linked to diseases in external studies (but are not yet backed by prospective clinical trials), do you wish to have access to this information? If you select 'No', this information will not be accessible to you, but will still be available to the data analysis team in anonymized form. Please note that you may be asked to disclose such information about disease markers or disease associations to insurance companies (e.g., before concluding new insurance contracts) or other institutions in the future.

☐Yes ☐No

12. Have you been diagnosed with a digestive disease?

☐Yes ☐No

13. In case we make a potentially interesting finding during the analysis of your sample, do you consent to be contacted by our data analysis team? This will make your email address available to the scientists performing the analysis and will allow them to ask you direct questions. All the scientists involved will have signed a **Non-disclosure Agreement** protecting your personal information.

☐Yes ☐No

**Clinical information (required)**

*Please answer the following questions only if you are completely sure. The more questions you answer the more fine-grained will be our analysis of your sample.*

1. What is your blood type? (select one)

☐ A+ ☐ A- ☐ B+ ☐ B- ☐ O+ ☐ O- ☐ AB+ ☐ AB-

2. Have you recently taken antibiotics in the last 2 months? ☐ Yes ☐No

3. Can you indicate your mode of birth? (select one)

a. How? ☐ Cesarean section ☐ Vaginal

b. Where? ☐ Hospital/Clinic ☐ Home

4. Can you indicate if you were breastfed during your infancy?

☐ Yes ☐ No

If yes, can you indicate how long? (select one)

☐ < 1 week ☐> 1 week ☐>1 month ☐ >1 year

5) Can you indicate to which category of the Bristol Stool Chart you would allocate your usual stool? (select one)

Type: ☐1 ☐ 2 ☐3 ☐4 ☐5 ☐6 ☐7

6) Can you indicate your average stool frequency? (select one)

☐ >twice daily ☐once daily ☐once every 2 days ☐< once every 2 days

7) If you are a women, can you indicate:

a. if you are pregnant: ☐Yes ☐No

b. if your period/menstrual cycle is regular ☐Yes ☐No

**Voluntary dietary information**

*Please answer the following questions only if you are completely sure. The more questions you answer the more detailed will be our analysis of your sample.*

1) Do you currently drink alcoholic beverages?

☐ Yes ☐ No, but I used to ☐ No, never drank alcohol

If yes, how often do you drink? (one drink is defined as 1 glass of beer, a shot of liquor, or 1 glass of wine, select one)

☐ Less than 1 drink per month ☐ 1-4 drinks per month ☐ 1-6 drinks per week ☐ 1-2 drinks per day ☐ 3 or more drinks per day

2) Do you usually take any probiotics? ☐ Yes ☐ No

3) How many portions of fruit and vegetable do you eat per day on average?

☐ less than 1 portion ☐ between 2 and 3 ☐ between 3 and 4

☐ 5 or more

**Data sharing**

*Sharing your metadata within our network will increase the scope of our study.*

Do you want to receive your personalised results (free of charge) to be able to monitor some study aspects and learn more about you own microbiota?

☐Yes ☐No

If **yes**, then please specify whether you are willing to share personal info to connect with others:

1. Do you want to anonymously share your digestive disease information in our network?

☐Yes ☐No

2. Do you want to anonymously share your voluntary information in our network?

☐Yes ☐No

3. Do you want to be contacted by others who have similar microbial profiles?

☐Yes ☐No

If you wish to share additional information or if you have a special request, please fill the box below:
