# Supplementary material for: The gut mycobiota of rural and urban individuals is shaped by geography
Source: BMC Microbiol. 2020 Aug 17;20:257. doi: 10.1186/s12866-020-01907-3 (PMC7430031; doi:10.1186/s12866-020-01907-3)
Supplement: Supplementary file 2 — Additional file 2. Results from the partition of variance analysis in RDA [file 12866_2020_1907_MOESM2_ESM.docx]

**Additional File 2 Results from the partition of variance analysis in RDA**

Call: varpart(Y = trflp_rda1_norm, X = assaychem_rda1_norm$Location,

assaychem_rda1_norm$Birthmode, assaychem_rda1_norm$Smoker,

assaychem_rda1_norm$Breastfeed, data = assaychem_rda1_norm)

Explanatory tables:

X1: assaychem_rda1_norm$Location

X2: assaychem_rda1_norm$Birthmode

X3: assaychem_rda1_norm$Smoker

X4: assaychem_rda1_norm$Breastfeed

No. of explanatory tables: 4

Total variation (SS): 86.801

Variance: 0.92342

No. of observations: 95

Partition table:

Df R.square Adj.R.square Testable

[aeghklno] = X1 1 0.02694 0.01647 TRUE

[befiklmo] = X2 1 0.01241 0.00179 TRUE

[cfgjlmno] = X3 1 0.01380 0.00320 TRUE

[dhijkmno] = X4 1 0.01556 0.00497 TRUE

[abefghiklmno] = X1+X2 2 0.03940 0.01852 TRUE

[acefghjklmno] = X1+X3 2 0.04011 0.01924 TRUE

[adeghijklmno] = X1+X4 2 0.04202 0.02120 TRUE

[bcefgijklmno] = X2+X3 2 0.02649 0.00533 TRUE

[bdefhijklmno] = X2+X4 2 0.02789 0.00676 TRUE

[cdfghijklmno] = X3+X4 2 0.03084 0.00977 TRUE

[abcefghijklmno] = X1+X2+X3 3 0.05280 0.02157 TRUE

[abdefghijklmno] = X1+X2+X4 3 0.05435 0.02318 TRUE

[acdefghijklmno] = X1+X3+X4 3 0.05711 0.02602 TRUE

[bcdefghijklmno] = X2+X3+X4 3 0.04347 0.01194 TRUE

[abcdefghijklmno] = All 4 0.06966 0.02831 TRUE

Individual fractions

[a] = X1 | X2+X3+X4 1 0.01637 TRUE

[b] = X2 | X1+X3+X4 1 0.00229 TRUE

[c] = X3 | X1+X2+X4 1 0.00513 TRUE

[d] = X4 | X1+X2+X3 1 0.00674 TRUE

[e] 0 -0.00012 FALSE

[f] 0 -0.00031 FALSE

[g] 0 0.00004 FALSE

[h] 0 -0.00013 FALSE

[i] 0 0.00004 FALSE

[j] 0 -0.00208 FALSE

[k] 0 -0.00008 FALSE

[l] 0 -0.00007 FALSE

[m] 0 0.00002 FALSE

[n] 0 0.00044 FALSE

[o] 0 0.00002 FALSE

[p] = Residuals 0 0.97169 FALSE

Controlling 2 tables X

[ae] = X1 | X3+X4 1 0.01625 TRUE

[ag] = X1 | X2+X4 1 0.01641 TRUE

[ah] = X1 | X2+X3 1 0.01624 TRUE

[be] = X2 | X3+X4 1 0.00217 TRUE

[bf] = X2 | X1+X4 1 0.00198 TRUE

[bi] = X2 | X1+X3 1 0.00233 TRUE

[cf] = X3 | X1+X4 1 0.00483 TRUE

[cg] = X3 | X2+X4 1 0.00518 TRUE

[cj] = X3 | X1+X2 1 0.00305 TRUE

[dh] = X4 | X2+X3 1 0.00661 TRUE

[di] = X4 | X1+X3 1 0.00678 TRUE

[dj] = X4 | X1+X2 1 0.00466 TRUE

Controlling 1 table X

[aghn] = X1 | X2 1 0.01673 TRUE

[aehk] = X1 | X3 1 0.01604 TRUE

[aegl] = X1 | X4 1 0.01622 TRUE

[bfim] = X2 | X1 1 0.00204 TRUE

[beik] = X2 | X3 1 0.00213 TRUE

[befl] = X2 | X4 1 0.00179 TRUE

[cfjm] = X3 | X1 1 0.00277 TRUE

[cgjn] = X3 | X2 1 0.00354 TRUE

[cfgl] = X3 | X4 1 0.00480 TRUE

[dijm] = X4 | X1 1 0.00472 TRUE

[dhjn] = X4 | X2 1 0.00497 TRUE

[dhik] = X4 | X3 1 0.00657 TRUE

---

Use function ‘rda’ to test significance of fractions of interest
