# Supplementary material for: The gut mycobiota of rural and urban individuals is shaped by geography
Source: BMC Microbiol. 2020 Aug 17;20:257. doi: 10.1186/s12866-020-01907-3 (PMC7430031; doi:10.1186/s12866-020-01907-3)
Supplement: Supplementary file 3 — Additional file 3: Fig. S1. Rarefaction plot showing sequencing coverage. The estimated average sequence coverage of high-quality paired end reads after quality control assessed using Nonpareil (in alignment mode). [file 12866_2020_1907_MOESM3_ESM.docx]

**Additional File 3 Figure S1** Rarefaction plot showing sequencing coverage. The estimated average sequence coverage of high-quality paired end reads after quality control assessed using Nonpareil (in alignment mode).
