# Supplementary material for: The gut mycobiota of rural and urban individuals is shaped by geography
Source: BMC Microbiol. 2020 Aug 17;20:257. doi: 10.1186/s12866-020-01907-3 (PMC7430031; doi:10.1186/s12866-020-01907-3)
Supplement: Supplementary file 4 — Additional file 4: Fig. S2. Venn diagram showing the unique and shared phylotypes for samples collected from urban and rural participants. [file 12866_2020_1907_MOESM4_ESM.docx]

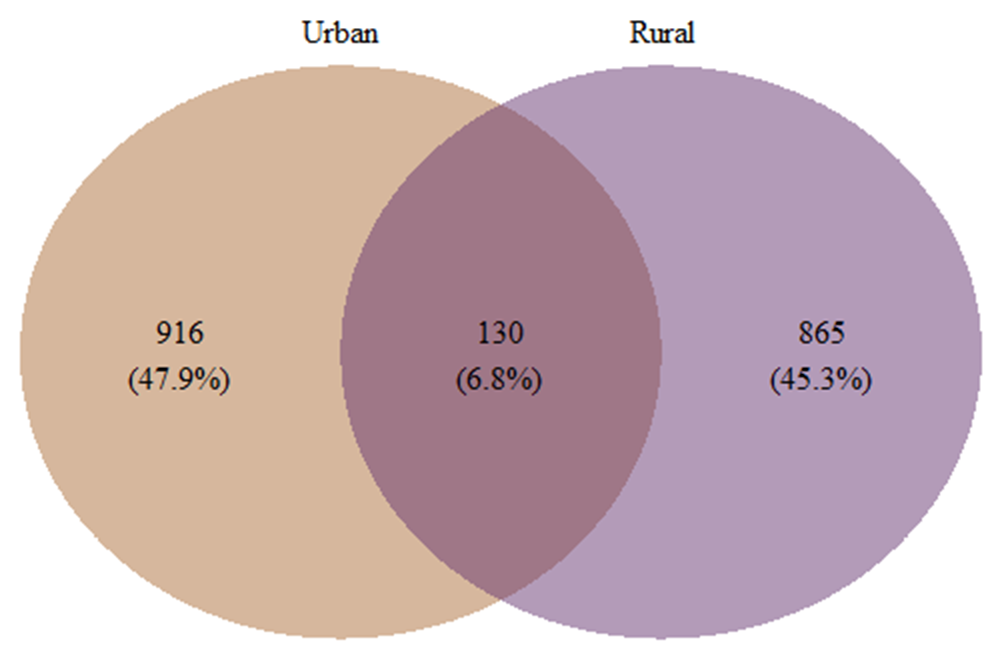


**Additional File 4 Figure S2**: Venn diagram showing the unique and shared phylotypes for samples collected from urban and rural participants.
