# Supplementary material for: The gut mycobiota of rural and urban individuals is shaped by geography
Source: BMC Microbiol. 2020 Aug 17;20:257. doi: 10.1186/s12866-020-01907-3 (PMC7430031; doi:10.1186/s12866-020-01907-3)
Supplement: Supplementary file 5 — Additional file 5: Fig. S3. Taxa abundance data was normalised to obtain the proportion of most abundant taxa per sample. The diameter of the points at the bottom of the plot corresponds to the magnitude of the LCBD value for a particular sample. The bars correspond to taxa that are most abundant with the top taxa sharing a bigger portion of the bar for each sample. [file 12866_2020_1907_MOESM5_ESM.docx]

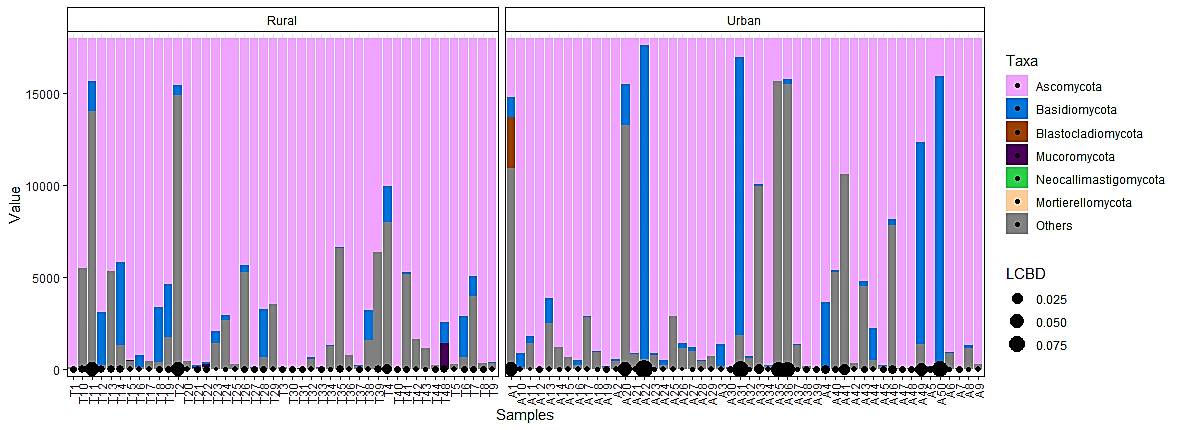


**Additional File 5 Figure S3** Taxa abundance data was normalised to obtain the proportion of most abundant taxa per sample. The diameter of the points at the bottom of the plot corresponds to the magnitude of the LCBD value for a particular sample. The bars correspond to taxa that are most abundant with the top taxa sharing a bigger portion of the bar for each sample.
